# Protein Misfolding and Aggregation: The Relatedness between Parkinson’s Disease and Hepatic Endoplasmic Reticulum Storage Disorders

**DOI:** 10.3390/ijms222212467

**Published:** 2021-11-18

**Authors:** Francisco J. Padilla-Godínez, Rodrigo Ramos-Acevedo, Hilda Angélica Martínez-Becerril, Luis D. Bernal-Conde, Jerónimo F. Garrido-Figueroa, Marcia Hiriart, Adriana Hernández-López, Rubén Argüero-Sánchez, Francesco Callea, Magdalena Guerra-Crespo

**Affiliations:** 1 Neurosciences Division, Cell Physiology Institute, National Autonomous University of Mexico, Mexico City 04510, Mexico; franciscopadilla@ifc.unam.mx (F.J.P.-G.); rramos@ifc.unam.mx (R.R.-A.); hmartinez@ifc.unam.mx (H.A.M.-B.); danielbernalconde@hotmail.com (L.D.B.-C.); jeros@ciencias.unam.mx (J.F.G.-F.); mhiriart@ifc.unam.mx (M.H.); 2 Regenerative Medicine Laboratory, Department of Surgery, Faculty of Medicine, National Autonomous University of Mexico, Mexico City 04510, Mexico; adriana.hernandez@unam.mx (A.H.-L.); rubenarguero@gmail.com (R.A.-S.); 3 Department of Histopathology, Bugando Medical Centre, Catholic University of Healthy and Allied Sciences, Mwanza 1464, Tanzania; francesco.callea46@gmail.com

**Keywords:** protein misfolding, protein aggregation, alpha-synuclein, Parkinson’s disease, endoplasmic reticulum storage disease, alpha-1-antitrypsin, alpha-1-antitrypsin deficiency, fibrinogen, hereditary hypofibrinogenemia with hepatic storage

## Abstract

Dysfunction of cellular homeostasis can lead to misfolding of proteins thus acquiring conformations prone to polymerization into pathological aggregates. This process is associated with several disorders, including neurodegenerative diseases, such as Parkinson’s disease (PD), and endoplasmic reticulum storage disorders (ERSDs), like alpha-1-antitrypsin deficiency (AATD) and hereditary hypofibrinogenemia with hepatic storage (HHHS). Given the shared pathophysiological mechanisms involved in such conditions, it is necessary to deepen our understanding of the basic principles of misfolding and aggregation akin to these diseases which, although heterogeneous in symptomatology, present similarities that could lead to potential mutual treatments. Here, we review: (i) the pathological bases leading to misfolding and aggregation of proteins involved in PD, AATD, and HHHS: alpha-synuclein, alpha-1-antitrypsin, and fibrinogen, respectively, (ii) the evidence linking each protein aggregation to the stress mechanisms occurring in the endoplasmic reticulum (ER) of each pathology, (iii) a comparison of the mechanisms related to dysfunction of proteostasis and regulation of homeostasis between the diseases (such as the unfolded protein response and/or autophagy), (iv) and clinical perspectives regarding possible common treatments focused on improving the defensive responses to protein aggregation for diseases as different as PD, and ERSDs.

## 1. Introduction

Most proteins have a characteristic three-dimensional structure determined by their amino acid sequence and thermodynamic factors [1]. Nonetheless, under the dysfunction of cellular homeostasis, proteins can adopt non-native spatial configurations, a process called misfolding [2]. Such misfolded proteins acquire conformations prone to polymerization into pathological aggregates, which may eventually cause pathologies [3,4]. This abnormal process is associated with a wide spectrum of disorders of the human body, among which neurodegenerative diseases stand out. Although each condition involves specific affected proteins, the formation of cellular protein inclusions is a common factor in most of them. Parkinson’s disease (PD), the second most common neurodegenerative disorder, involves the misfolding of a protein named alpha-synuclein (α-syn), whose aggregation generates inclusions known as Lewy bodies (LBs) that can be found in neurons [5]. Similarly, this misfolding pattern is observed in other disorders of the central nervous system such as Alzheimer’s disease (AD, where the aggregation is caused, among other factors, by the pathological amyloid precursor protein) and Huntington’s disease (HD, where the aggregated protein in the brain is huntingtin) [4,6].

Notwithstanding, albeit being distinctive of several neurodegenerative disorders with similar pathophysiological processes, protein misfolding and aggregation is not an exclusive phenomenon, as it has also been observed in other unrelated conditions that might at first glance be thought to be unrelated. An example of this is endoplasmic reticulum (ER) storage disorders (ERSDs), systemic diseases whose main pathological hallmark is the misfolding and aggregation of proteins such as alpha-1-antitrypsin (AAT) and fibrinogen (FG) in hepatocytes [7,8]. Likewise, other extrahepatic conditions associated with protein misfolding exist, e.g., cardiac atrial amyloidosis, cystic fibrosis, and sickle cell disease. In fact, accumulating evidence suggests that, as more advanced genetic and proteomic techniques are developed, more protein misfolding diseases could be detected [9]. On this basis, it is necessary to deepen our understanding of the basic principles of misfolding and aggregation common to these diseases, which, whilst heterogeneous in symptomatology, share the initial pathophysiological processes, which suggests the possibility of developing common treatments focused on recovering the homeostasis lost due to this aggregation.

In view of the above, throughout this review, we provide a comprehensive comparison of a neurodegenerative disease, PD, and its associated aggregative protein, α-syn, with two non-neurodegenerative ERSDs: AAT deficiency (AATD) and hereditary hypofibrinogenemia with hepatic storage (HHHS). We briefly cover their pathogenic characteristics to better comprehend the biochemical and molecular bases that lead to similar aggregative phenomena between such different proteins that do not appear to have shared features. Furthermore, we compare the defensive stress mechanisms that take place upon protein misfolding and aggregation, especially in the ER, which is known to suffer stress in different organs in the quest to conserve homeostasis and preserve cell survival. All of the above aims to provide a frame of reference that will allow the future development of possible common treatments focused on improving proteolytic mechanisms related to the degradation of proteins no longer susceptible to excretion/degradation in these diseases by identifying similarities in their pathophysiological processes. It should be noted that the choice of liver conditions for comparison with a neurodegenerative disease, over other more related diseases such as AD or HD, aims precisely to highlight the possible coincidences between pathologies that until now were considered distant, which could lead to an approach to common treatments for diseases with common basic molecular mechanisms.

## 2. Alpha-Synuclein

### 2.1. Alpha-Synuclein Aggregation Induces Parkinson’s Disease

**Figure 1:**
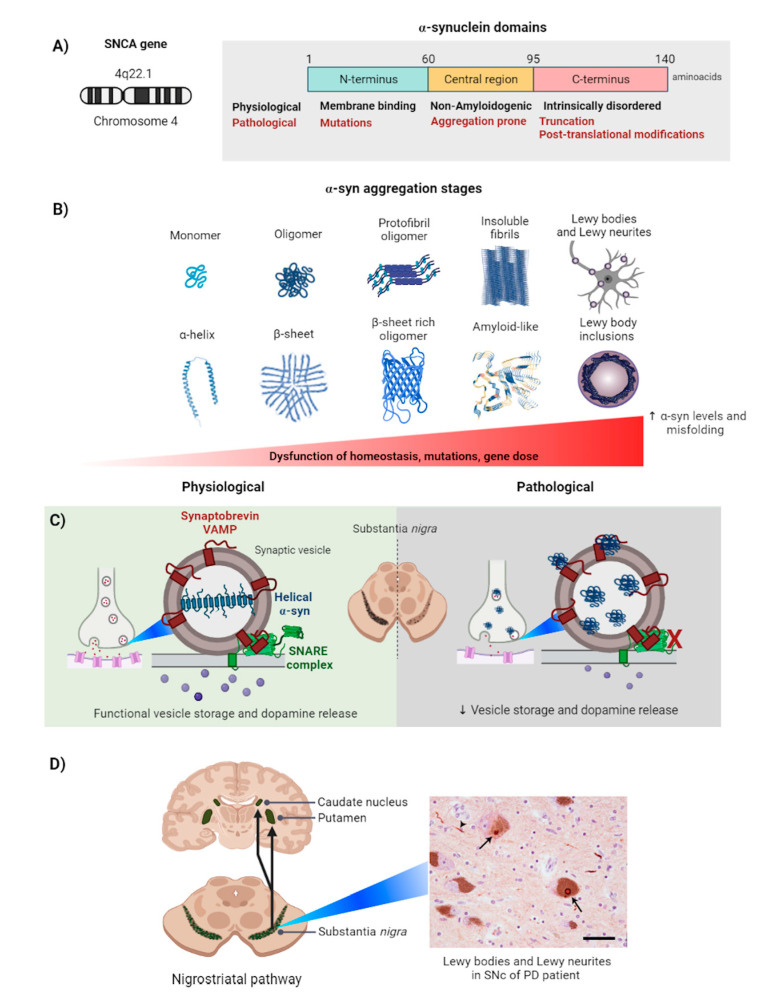
Misfolding and aggregation of α-syn in PD. (**A**) Left: *SNCA* gene coding for the α-syn on chromosome 4. Right: Structure of α-syn (Protein Data Base, PDB, 1XQ8) highlighting its different domains and its involvement in cell physiology (black) as well as in various alterations that come to affect its function (red) [17]. (**B**) Aggregation states of α-syn: structure (above) and three-dimensional composition (PDB 6OSJ) (below) by crystallography. (**C**) α-syn activity under physiological conditions (left), as well as in alterations to its function (right). Image modified from Burré et al. (2010) [18]. (**D**) Left: Nigrostriatal pathway, which originates in the SNpc and projects to the dorsal striatum. Right: α-syn inclusions in the form of LBs (arrows) and neurites (arrowheads) from a PD patient (20×). Scale = 50 μm. Image taken from Ingelsson (2016) [19].

α-Syn is a 15 kDa protein encoded by the *SNCA* gene, which is located in the long arm of chromosome 4 (Chr 4q22.1) (Figure 1A) [10]. This protein is composed of 140 amino acids distributed over three domains. The first region, the *N*-terminal, is composed of 4 regions of 11 “imperfect” repeats with a lysine-rich consensus sequence (KTKGEV) [11]. This sequence is relevant for α-helix conformation in the α-syn protein, facilitating its binding to negatively charged lipids [3,10]. However, this domain is characteristically vulnerable to missense mutations (e.g., A53T, A30P, E46K) with different phenotypes and pathogenic mechanisms in PD [12]. Then follows the central region, referred to as the “non-amyloidogenic component” (NAC), known for its hydrophobic properties that becomes prone to aggregation under specific conditions, being responsive to conformational changes in α-syn, from random coiled-coils to β-sheet structures in an oligomeric state [13,14]. The last region is the carboxyl-terminal region, which is characterized by acidic residues which confer to α-syn an intrinsically developed structure mediating protein-protein interactions [11]. This domain may present alterations in its structure such as truncation, which consists in the elimination of acidic residues promoting the aggregation of α-syn into fibrils [15]. Related to this, post-translational modifications may occur in the C-terminal domain, such as phosphorylation (serine 129 and 87), ubiquitination (E3 ubiquitin-ligases), nitration (tyrosine residues: Y39, Y125, Y133, and Y136), and O-GlcNAcylation (threonine residues: T33, T34, T54, and T59) that influence α-syn aggregation [16].

The native structure of α-syn remains under investigation however, recent evidence describes it with an intrinsically unfolded structure [20,21], in an α-helix structure [22,23], or a combination of both [18] (Figure 1B). The structural plasticity of α-syn is dependent on cellular homeostasis and interactions with other cellular components. For instance, it has been reported that the presence of membrane phospholipids favors the α-helix structure in α-syn [24,25]. The functional role that this interaction could have still requires further research. Additionally, there are factors, such as variations in pH, salt concentration, and lipid composition, that can modify the structure of the α-syn [26]. Likewise, changes in the protein composition of α-syn have been reported, such as phosphorylation, glycosylation, and acetylation that contribute to structural and functional changes [11,21,27].

Under physiological conditions, α-syn is considered to promote membrane curvature in presynaptic dopaminergic terminals, contributing to vesicle formation and vesicular trafficking [28,29] (Figure 1C). These functions are enhanced by its association with the SNARE complex consisting of synaptobrevins, vesicular membrane-associated proteins (VAMP), syntaxins, and synaptosome-associated proteins (SNAP25) [30]. This interaction suggests a potential role for α-syn in the regulation of dopamine release. Furthermore, the α-syn can adopt other structural conformations under certain physiological or pathological conditions that have begun to be elucidated. 

Dysfunction of cellular homeostasis and/or mutations in the *SNCA* gene can induce misfolding of the native structure of α-syn, leading to loss of structure-associated functions and its aggregation (as will be described later on). This abnormal accumulation of α-syn, as well as its aberrant conformation in neurons and glia, leads to neurodegenerative diseases known as synucleinopathies [31], such as PD, pure autonomic failure, multiple system atrophy (MSA), and dementia with LBs (DLB). For this review, the focus will be exclusively on PD.

Accounting for up to 15% of all cases of dementia, PD is the second most common cause of neurodegeneration after Alzheimer’s disease [32]. PD affects people with an average age of 55 years and manifests with physical and neuropsychiatric symptoms. Physical symptoms are mainly motor, such as slow and imprecise movements (bradykinesia), tremor at rest, decreased facial expression (hypomimia), difficulty walking, freezing, and postural imbalance [32]. Neuropsychiatric alterations involve cognitive deterioration, dementia, impulse control disorder, apathy, depression and anxiety, psychosis, and hallucinations [32]. PD is mainly, but not exclusively, attributed to the death of dopaminergic neurons in the substantia nigra pars compacta (SNpc), a region located in the midbrain. These neurons project to the dorsal striatum, forming the nigrostriatal pathway. Dopaminergic signaling in the SNpc regulates movement coordination through its communication with the basal ganglia, and muscle contraction through its association with the spinal cord. Disruption of dopaminergic neurons in this structure compromises dopaminergic signaling, causing the characteristic motors symptoms of PD [33]. 

More than 200 years after its description, the etiology of PD remains unknown. However, genetic, and environmental factors involved in the neuropathology of the disease have been identified [33]. Among the most important genetic factors is the *SNCA* gene, which encodes for α-syn. Alterations in the *SNCA* gene include mutations or increased gene dosage, such as duplications and triplications [34]. The aforementioned increases α-syn expression and toxicity, making it critical to understand the structure and function of α-syn in the evolution of the disease. PD is characterized by the development of cytoplasmic inclusions in dopaminergic neurons named Lewy bodies (LBs). The presence of these inclusions is one of the main pathological features in the brain biopsies of PD patients, which consequently has been associated as the cause of familial PD [35,36]. In support of this, increased gene dosage, as well as autosomal dominant mutations in the *SNCA* gene, lead to the early onset of PD. While the aim of this article is to review the intracellular mechanisms of α-syn leading to cellular damage, a proper compilation of advances in synucleinopathies requires an adequate acknowledgement of the recent findings on the mechanisms of α-syn transmission, as they may shed light on the possible origin of PD and other related pathologies. Among them, the prion-like propagation theory (elegantly reviewed by Jan et al. [37]) is increasingly accepted with new studies demonstrating its transmission between neurons and its trans-synaptic propagation from the peripheral nervous system to the brain via the sensory or enteric nervous systems. For instance, Ferreira et al. [38] observed that peripheral inoculation of preformed α-syn fibrils in a mouse model of PD derived in a trans-synaptic and retrograde propagation, demonstrating the prion-like propagation mechanism in which aggregates are directly transferred between neurons and act as a seed for the generation of new aggregates in recipient cells. Similarly, Van Den Berge et al. [39] evaluated and demonstrated bidirectional spread of α-syn aggregates through the vagus nerve, i.e., from the duodenum to the brainstem and stomach. Whether similar mechanisms exist in ERSDs remains to be determined.

### 2.2. Alpha-Synuclein Aggregation in the Cell

The α-syn is present in various conformations within the cell, from its physiological conformation of soluble monomers to pathological oligomers and fibrils formed by aggregation processes [18] (Figure 1B). When misfolded into fibrils, α-syn adopts a crossed β-sheet conformation, whose properties confer on it the classification of an amyloidogenic protein [40]. Amyloid formation in α-syn involves three types of polymers: dimers, oligomers, and fibrils [41]. Initially, the clustering of monomers of α-syn leads to the formation of dimers. These aggregates form oligomeric structures, which in turn group into fibrillar clusters [42]. Oligomers are considered an important conformation in the fibrillar process, acting as a structural core in the increased aggregation of α-syn [41].

Recently, the pathological function of α-syn has begun to be elucidated from its structural features (Figure 1C). In 2016, the fibrillar structure of α-syn was first observed in detail, demonstrating that it is rich in β-sheets. Using solid-state nuclear magnetic resonance and cryo-electron microscopy techniques [43,44,45], the native structure of α-syn was described as a single 5 nm protofilament, or as a dimerized 10 nm filament. Both structures have been observed in samples extracted from the SNpc of PD patients [43,44,45]. On the other hand, it was possible to determine that the dimeric α-syn filament is a more mature type of fibril than the protofilament. These fibrils present hydrophobic residues flanked by strong “ionic locks” forming electrostatic interactions at the core of the fibril. This process potentially increases the energetic contribution of the fibril in aggregates [43,44,45]. 

Related to the above, Roostaee and colleagues, showed that dimerization of α-syn can accelerate transformation to oligomers, suggesting that dimerization could also be an important step in the initiation of the fibrillation process [46]. In addition, other studies have found that mutations in *SNCA* (e.g., A53T), duplications or triplications, increased oxidative stress, and environmental stressors could induce or increase α-syn aggregation and toxicity [47,48]. In support of this, several in vitro studies propose that the aggregation pathway for α-syn amyloid fibril formation depends on nucleated polymerization, that is, aggregation begins with a primary nucleation of monomers on the surface of the lipid membrane, followed by elongation of fibrils by addition of monomers, and, subsequently, secondary nucleation of monomers occurs on the surface of already existing fibrils [24,47,48].

Misfolding of α-syn to fibrils that make up LBs requires alterations in homeostasis and folding pathways. Relatedly, emerging evidence suggests that oligomers represent the toxic species leading to PD [49,50]. The cytotoxic effects of fibrils of α-syn have been linked to increased oxidative stress, impaired axonal transport, impaired ubiquitin–proteosome machinery, mitochondrial function, and synaptic dysfunction [51]. The stimuli that trigger oligomer formation are still unknown, although it has been observed that changes in pH and temperature of the medium may contribute to this process [52]. Furthermore, considering the role of α-syn in presynaptic terminals at the pathological level the question of its transmission and propagation for the formation of LBs arises. In this regard, it is known that pathological α-syn aggregates are distributed in an anterograde and retrograde manner, accelerating the spread of cytotoxic α-syn, and thus, neurodegeneration to the whole brain [53].

Conversely, although usually associated to a pathophysiology process, aggregation of α-syn in mature fibrils could also be interpreted as a neuroprotective measure against the formation of soluble oligomers, this to reduce the toxicity of the number of exposed β-sheets, which induce further aggregation of α-syn. Thus, as the formation of toxic oligomers is inhibited, fibril formation is blocked. On the other hand, recent research questions whether experimentally preventing or inhibiting fibril formation could have a counterproductive effect, i.e., fragmenting fibrils could extend the lifetime of the oligomers. This suggest the possibility that future research should focus on an intermediate point of oligomer and fibril formation to stabilize the α-syn structure and inhibit the progression of its aggregation [50].

With these findings, new evidence is unveiled on the structural changes of α-syn that precede its aggregation during PD development. In turn, these data provide new insights into the folding and formation of the native and pathogenic conformations of the α-syn protein.

### 2.3. Physiological Response to α-Syn Aggregation: Autophagy and Proteosomes

The autophagy–lysosome (ALP) system is responsible for degrading a-syn, along with other proteins and even cellular organelles. The ALP is composed of the macroautophagy, chaperone-mediated autophagy (CMA), and microautophagy pathways, which transfer intracellular components to lysosomes. The latter are responsible for degrading or recycling proteins, plasma membrane constituents and other extracellular material [54]. Indeed, evidence obtained from post mortem samples of humans, transgenic mice, and cellular models of PD, have related alterations in ALP with the accumulation of α-syn. Similarly, it has been reported that multiplications, mutations, and post-translational modifications of the protein further impair the function of autophagy pathways, generating a vicious cycle that leads to neuronal death [55]. Macroautophagy and CMA are the two ALP pathways involved in α-syn degradation [56,57]. 

Macroautophagy involves the degradation of a-syn through the formation of autophagosomes. These fuse with lysosomes, forming autolysosomes. In PD, α-syn aggregates impair macroautophagy by reducing autophagosome clearance, which may contribute to the increased death of dopaminergic neurons in advanced stages of the disease [58]. Indeed, conditional deletion of the expression of the macroautophagy gene ATG-7 in dopaminergic neurons leads to cell death and a decrease in striatal dopamine levels. In turn, this suppression triggers the formation of ubiquitinated protein aggregates, positive for p62 and ubiquitin [59]. It also causes the accumulation of α-syn in striatal dopaminergic terminals [60]. The latter is consistent with the physiological function of α-syn at presynaptic terminals and, in turn, with the role of macroautophagy in axonal processes [61]. From another point of view, pharmacological inhibition of macroautophagy with 3-methyladenine (3-MA), leads to the accumulation of both endogenous and overexpressed α-syn [56]. Interestingly, in vitro induced macroautophagy decreases the overexpression levels of wild-type (WT) and mutant α-syn [62]. 

Nonetheless, as mentioned above, α-syn alterations also impair macroautophagy. For instance, in mammalian cells and transgenic mice, overexpression of α-syn WT and the A30P and A53T mutations lead to inhibition of macroautophagy [63,64]. This is due to a reduction in the formation of autophagosomes [63], inhibiting the RAB1A protein, a GTPase involved in early secretory pathways, causing a mislocalization of the early autophagy protein ATG-9 and reducing omegasome formation [63], an autophagic structure that is frequently observed in association with ER [65]. Likewise, mutant α-syn expression promotes morphological and functional abnormalities in the autophagolysosomal system, preventing lysosomal fusion of autophagosomes and reducing the removal of both α-syn itself and dysfunctional mitochondria through mitophagy [66]. Finally, posttranslational modifications of α-syn, such as phosphorylation and SUMOylation, accelerate its turnover through macroautophagy, a process conserved from yeasts to mammals [67,68]. 

Taken together, this evidence shows that there are alterations of α-syn following macroautophagy impairment, suggesting that this pathway regulates α-syn turnover. Furthermore, macroautophagic degradation of α-syn appears to be conformationally dependent or accelerated under conditions of overexpression and mutations, although these processes need to be determined in vivo.

CMA, the second autophagic pathway observed in PD, is a highly selective catabolic process that, unlike macroautophagy, does not involve vesicle formation. Instead, substrates directly cross the lysosomal membrane to reach the lysosomal lumen. The CMA is a specific process because only cytosolic proteins having a CMA-related targeting motif (KFERQ) are recognized by a chaperone complex involving the 70 kDa heat shock protein 8 (Hsc70). With this recognition they translocate to the lysosome to interact with the lysosome-associated membrane protein type 2A (LAMP2a) to degrade the components by hydrolytic enzymes [69]. In human neuronal lines and primary neuronal cultures, the CMA pathway degrades α-syn WT [56,69]. Inhibition of CMA leads to the formation of α-syn oligomers although confirmation with in vivo experiments is required. However, unlike macroautophagy, the CMA pathway apparently only degrades α-syn monomers and dimers. In post mortem investigation of patients with PD, the heat shock protein (Hsc70) and the lysosome-associated membrane protein 2a (LAMP2a), both necessary for the CMA pathway [70,71], are significantly decreased. This correlates directly with increased α-syn levels and the accumulation of cytosolic substrates of the pathway [72]. Furthermore, as observed for macroautophagy, CMA is also impaired due to mutations (A30P and A53T inhibits it [73,74,75]) and posttranslational modifications (oxidation and nitration of α-syn decrease its degradation, whilst phosphorylation almost completely enables it [74]).

As mentioned in the previous paragraphs, macroautophagy and CMA are impaired in PD, and both autophagic pathways alterations also contribute to the progressive propagation of misfolded α-syn. This occurs through repeated cycles of release and uptake of both oligomers and α-syn fibrils, leading to prion-like propagation [76,77]. 

To further highlight the relationship between α-syn and ALP pathways, there is evidence supporting a direct relationship between α-syn and the lysosomal enzyme β-glucocerebrosidase (GCase). In line with this, two studies showed decreased GCase activity in cerebrospinal fluid of PD patients compared to controls [78,79]. Then, molecular or pharmacological dysregulation of GCase was described to promote α-syn aggregation in several cell models [80,81]. Furthermore, when impairments in the lysosomal enzyme are prolonged, it increases α-syn oligomerization and aggregation in various brain regions [82]. Over the years, it has been established that GCase activity and content decrease in SNpc and other brain regions in different studies of patients with sporadic PD [83,84]. Similarly, reduced activity of another lysosomal enzyme was found in blood samples from PD patients, α-galactosidase A [85]. This has also been reported in leukocytes from patients with sporadic PD [86]. This evidence shows a relationship between decreased activity and levels of different lysosomal enzymes with the development of PD.

In addition to autophagy, the ubiquitin-proteasome system (UPS) is another major pathway for the degradation of misfolded proteins in mammals, fundamental in the maintenance of cellular proteostasis. Misfolding of α-syn affects UPS function leading to increased Lewy body formation and culminating in neuronal death. This has been observed in vitro [87,88] and in vivo [89] models of PD. In addition, a decrease in proteasome catalytic activity has been observed in brain tissue from post mortem PD patients compared to healthy control samples [90,91]. This evidence suggests a direct effect of misfolded α-syn on UPS function in advanced stages of PD. On the other hand, experimental inhibition of the UPS pathway in vivo replicates the neuropathological features of PD [92]. Moreover, in a recent study, AAV-mediated overexpression of α-syn in rat SNpc dopaminergic neurons results in early accumulation of the proteasome-targeted protein Ub^G76V^, which is a signal of ubiquitin fusion degradation. This precedes UPS dysfunction and dopaminergic neurodegeneration, suggesting that accumulation of misfolded α-syn in vivo triggers UPS dysfunction in dopaminergic neurons, causing progressive cellular dysfunction and culminating in cell death due to proteostasis failure [89].

## 3. Alpha-1-Antitrypsin

### 3.1. Alpha-1-Antitrypsin Aggregation Induces Serpinopathies

**Figure 2:**
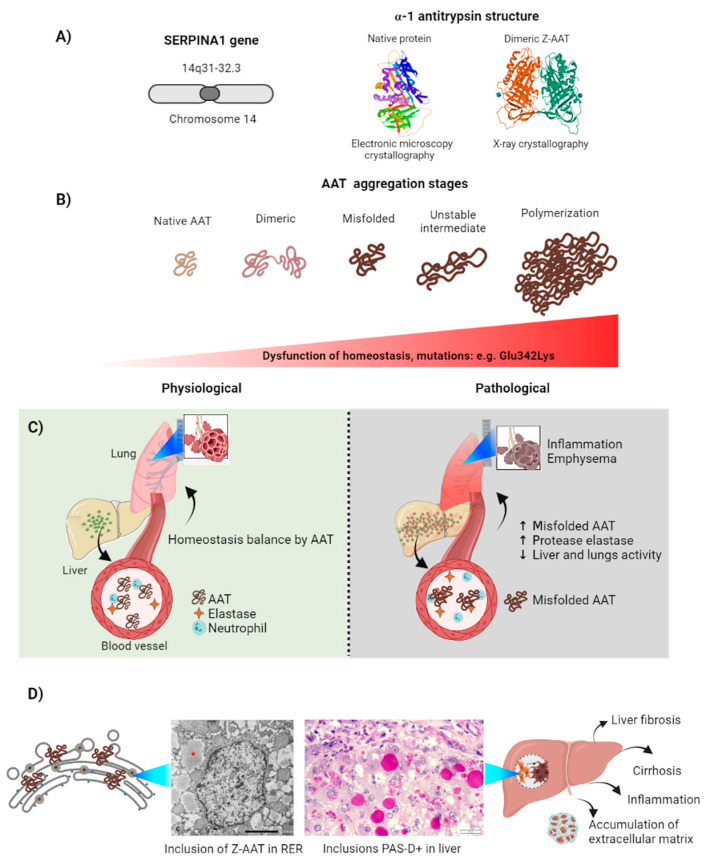
Z-AAT misfolding and aggregation in AAT Deficiency. (**A**) Left: Serpin-1 gene coding for AAT on chromosome 14 [99]; Right: Structural detail of native (PDB 3NE4) and dimeric Z-AAT (PDB 5IO1) structures obtained by crystallography. (**B**) Aggregation states of AAT. (**C**) AAT activity under physiological conditions (left) and in alterations to its function (right). (**D**) Left: Z-AAT aggregates in the ER in a liver sample from a patient with AATD. Next, an image taken by electronic microscopy (15,725×) showing a hepatocyte whose rough ER shows dilated cisternae with accumulated AAT. Image taken from Callea et al. (2021) [100]. Right: Liver tissue section with hepatocytes containing periodic acid-Schiff with diastase (PAS-D) positive inclusions, distinctive of AAT accumulation. Scale = 20 μm. Image taken from Callea et al. (2021) [100]. These affectations lead to the development of liver fibrosis, cirrhosis, prolonged inflammation, and extracellular matrix accumulation in liver and lungs [101].

AAT is a 52 kDa glycoprotein (394 amino acids) that acts as a plasma protease inhibitor [93]. Mainly synthesized within hepatocytes, pulmonary and intestinal alveolar cells, neutrophils, macrophages, and cornea, AAT’s main role is to protect lung tissue by inhibiting cathepsin G, serine proteases neutrophil elastase, and proteinase 3 [94,95]. AAT is encoded by the Serpin Family A Member 1 (*SERPINA1)* gene, translated in free ribosomes and translocated to the ER where it acquires its final conformation constituted of nine α-helices, two β-sheets, and a reactive center loop (Figure 2A) [96,97], after which it is exported to the bloodstream via the Golgi apparatus. Like all serine protease inhibitors, AAT’s characteristic secondary structure can suffer alterations due to mutations in the *SERPINA1* gene, which can lead to non-functional proteins that can polymerize and accumulate: these conditions are known as serpinopathies [98]. 

Over 75 mutations of the *SERPINA1* have been identified, many with clinically significant effects [102]. Particularly, a genetic variant of AAT known as the Z allele (Z-AAT), in which the homozygous substitution of a single amino acid (Glu342Lys) occurs due to a single base-pair substitution, generates a severe and common genetic disease known as AATD [103] (Figure 2B). This mutation promotes protein misfolding and its aggregation into polymeric chains of AAT within the ER in hepatocytes. As a consequence, this pathological process results in liver injury by impairing basal hepatocyte function, unlike the non-pathological M (wild type) allele, or the S (Glu264Val) allele that generates mild AATD [104] (Figure 2C). It is estimated that between 2% and 5% of Europeans exhibit the heterozygous M and Z alleles genotype, condition that also promotes heterogeneous intracellular polymer formation [105]. Both mild and severe AATD have been classified as serpinopathies. Common complications associated with AATD include chronic obstructive pulmonary disease (COPD), cirrhosis, neonatal jaundice, or panniculitis [106,107].

### 3.2. Z-AAT Aggregation in the Cell

Under normal conditions, AAT is initially synthesized in the free ribosomes and then translocated into the rough ER, where post-translational modifications take place to activate it and release it into the bloodstream (Figure 2B) [108]. However, in AATD, the Z-AAT mutation induces polymerization of AAT in the ER with either a M-AAT or another Z-AAT. This reduces plasma AAT levels and generates loss of function, ultimately damaging hepatic and alveolar cells (Figure 2B) [104]. Particularly, in severe AATD, the aggregation process leads to the formation of inclusion bodies that behave like matrices through which smaller proteins can diffuse. Interestingly, the ER remains partially functional even after fragmentation by Z-AAT [109]. Moreover, trafficking of such soluble proteins between Z-AAT inclusions requires cytosolic factors such as the secretion-associated Ras related GTPase 1A (Sar1) and sec22B, a member of the SEC22 family of vesicular proteins [109]. 

Although the above has been widely documented, the precise mechanism of polymerization in the ER remains largely unknown, mostly due to the heterogeneity of ex vivo polymers that makes them unsuitable for crystallography [110]. Nonetheless, the development of new optical and computational techniques is currently making it possible to obtain better representations of the structural conformation of Z-AAT in its aggregated form. Indeed, it has been reported that in the Z allele, Glu-342 is located at the top of strand 5 at the central β-sheet A (s5A) in the P17 position, which is the active site located at the base of the reactive center loop: this position allows Glu-342 to generate a salt bridge to Lys-290, as well as a hydrogen bond to Thr-203. This conformation forces the reactive loop into a hinge with Glu-342 [93,111]. Thus, a labile reactive loop from another AAT can be inserted into the β-sheet A, promoting polymerization [93]. 

Likewise, advances in organoid technology are helping to overcome the difficulty of analyzing human samples ex vivo. By culturing human liver organoids from ZZ homozygous patient cells, Gómez-Mariano and colleagues [112] were able to generate liver organoids. As expected, this model reproduced the Z-AAT aggregation observed in the liver, where 10% of the organoids were positive for the presence of Z-AAT polymer aggregation. In contrast, monomeric AAT protein was not detected in the cell extracts or in the extracellular medium of Z-AAT organoids [112], highlighting the relevance of understanding the mechanism of ZZ homodimer aggregation using models with alternative experimental capabilities.

Following the foregoing, it has recently been shown that only the Z allele is sufficient to form intracellular polymers in the ER [94]. Indeed, by labeling the MZ variant proteins in liver explants from heterozygous patients with an antibody specific for each allele, and localizing them through crystallography, Laffranchi and colleagues [94] found that M- and Z-AATs can polymerize together within the ER, indicating that Z-AAT can form heteropolymers with non-polymerizing variants in vivo. Furthermore, it appears that the polymer chains of hepatocytes from a MZ-AAT heterozygote contain a small percentage of M variants, which closely resembles ZZ polymers formed solely by proteins from the Z allele [94]. 

In parallel, Faull and colleagues [110] modeled various conformations of aggregated AAT. Using explanted livers of individuals homozygous for Z-AAT and recombinant proteins from *Escherichia coli*, they found that the open, linear dimeric 3D model (H4 *C*-terminal) was the most compatible with the 60° and 90° dimers present in liver-derived polymers (Figure 1D). These dimers have an opening angle between their c-loop of 60° and 90°, respectively. Indeed, the H4 *C*-terminal structure involves the displacement of the 4-kDa *C*-terminal fragment of Z-AAT, resulting in a flexible arrangement [110], in contrast to the previously proposed circular conformation of Z-AAT dimers [113]. Thus, their data support the idea that linear C-terminal domain swap is the structural basis for pathological polymers of Z-AAT [110].

### 3.3. Physiological Response to Z-AAT Aggregation: Autophagy and Proteosomes

Autophagy is the main pathway for Z-AAT degradation (Figure 2D). However, no consensus has been reached on the mechanisms of autophagy promoted by Z-AAT aggregation in the ER [114]. The most widely accepted general process indicates that autophagy is triggered by polymerized Z-AAT, which is introduced into autophagic vacuoles for its degradation [115]. As expected, Z-AAT autophagosomes are widely present in hepatocytes of AAT-deficient mice and patients, and Z-AAT degradation has been observed to be impaired by autophagy inhibitors [116,117,118]. Nonetheless, the clearance provided is insufficient, as a proportion of Z-AAT aggregates remains within inclusions, giving rise to liver damage and fibrosis [115,119]. In view of the above, several studies have shown that induction of autophagy reduced the presence of such conditions [114,120], so attention has been focused on the signaling pathways and proteins involved in the autophagy process in the presence of Z-AAT aggregation in the search to improve the response.

In this regard, Feng and colleagues [121] showed that the ubiquitin ligase SYVN1/HRD1 appeared to play a role in Z-AAT elimination by enhancing Z-AAT degradation through the autophagy–lysosome pathway. This clearance was impaired following autophagy inhibition, as well as in autophagy-related 5 knockout cells. They reported that inducing autophagy resulted in enhanced SYVN1-mediated Z-AAT degradation through ubiquitination, which is required for its autophagic degradation by enabling the interaction between Z-AAT and sequestosome-1/p62, an autophagy receptor required for the formation of the autophagy complex [121]. 

Similarly, Tang and colleagues [122] evaluated the molecular mechanism of the compound 24-nor-ursodeoxycholic acid (norUDCA) in the autophagy pathway of Z-AAT clearance. norUDCA is a drug that induces Z-AAT degradation by activating hepatic regulatory genes for autophagy [123]. Thus, they found that the AMP-activated protein kinase phosphorylates Unc-51 like autophagy activating kinase 1, an important protein that is involved in the early biogenesis of autophagosomes. This way, the phosphorylation at Ser317, Ser555, and Ser777, as well as the inhibition of Ser757, initiates autophagy, promoting the degradation of Z-AAT polymers and reducing their aggregation in hepatocytes.

Additionally, downstream targets of the NFκB signaling pathway have recently been shown to play a critical role in the autophagic disposal of misfolded proteins [117]. This may lead to better development of targets of autophagy signaling pathways to reduce the damage caused by Z-AAT polymerization. 

On the other hand, around the research to inhibit autophagy repression, Hidvegi and colleagues [124] found in livers of AATD patients that the levels of the regulator of G-protein signaling 16 (RGS16) were up-regulated and that it was capable of binding to the Gαi3 subunit of the heterotrimeric G protein Gi3. The Gαi3 subunit is known to regulate autophagy through the PI3K/protein kinase B/mTOR pathway during hepatic anti-autophagic action [125,126]. Therefore, they speculated that binding of Gαi3 to RGS16 might inhibit G signaling, and in doing so, depresses the autophagy response [127]. 

However, although not as important as the process of autophagy, another mechanism known to provide AAT clearance is the proteasome [128]. It has been documented that Z-AAT is degraded through the ER-associated protein degradation (ERAD) pathway, as the OS-9 protein and the ER chaperone GRP94 form a complex with Z-AAT and deliver it to the sel-1 protein homolog 1 and HRD1, which reduces its solubility, facilitating its removal by the proteasome [129,130,131]. Interestingly, the VPS30/ATG-6 genes of the ERAD pathway activate autophagy when ubiquitinated proteins are not degraded by the proteasome. Thus, when there are low levels of Z-AAT, the proteasome disposes them, but with higher levels of Z-AAT, autophagy is activated by VPS30/ATG-6 to degrade aggregated polymers [132]. Although the proteasome appears to have a lesser role in Z-AAT degradation than macroautophagy, further investigation of the interrelationship between these two mechanisms could allow a better understanding of the complete clearance pathway and the development of improved pharmacological strategies to reduce Z-AAT aggregation in the ER [128]. 

## 4. Fibrinogen

### 4.1. Fibrinogen Aggregation Induces Coagulopathies

**Figure 3:**
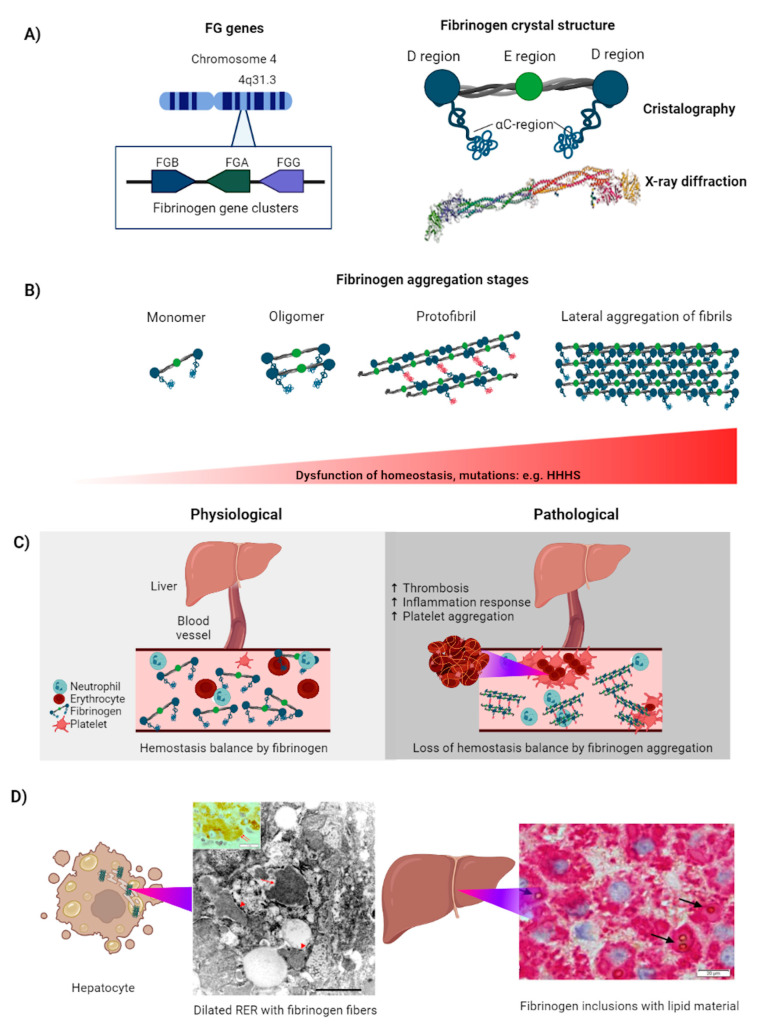
FG misfolding and aggregation in HHHS. (**A**) Left: FG gene coding for FG on chromosome 4 [136]. Right: Native structure of FG and its three-dimensional structure (PDB 3GHG). (**B**) Aggregation states of FG. (**C**) FG activity under physiological conditions (left) and alterations in its function under HHHS (right). (**D**) Histopathological damage caused by HHHS. Left: Electronic microscopy (8000×) of hepatocyte showing dilated rough ER and aggregation of FG into tubular structures or elongated fibers. Image taken from Callea et al. (2021) [100]. Right: Section of liver tissue from a patient with HHHS. Hepatocytes contain FG immunoreactive inclusions (red) with apolipoprotein B (arrows) positive lipid material (original magnification of 60×). Image taken from Callea and Desmet (2021) [137].

FG is a 340 kDA glycoprotein synthesized in the liver and normally found in circulating blood as a covalently linked hexamer [133,134] (Figure 3A). It is involved in several key processes related with the acute phase response caused by tissue injury, such as the hemostatic cascade, fibrinolysis, inflammation, and angiogenesis [135]. Its structure consists of 2 heterotrimers, composed of polypeptide chains Aα, Bβ, and γ [133]. Each chain is joined by disulfide bonds, with a central E region connected to two globular D regions [135]. FG chains are coded by the FG α-chain (*FGA*), FG β-chain (*FGB*), and FG γ-chain (*FGG*) genes in chromosome 4q31.3 [134]. Although expressed mainly in the liver, FG transcripts can also be found in the stomach, lungs, kidneys, and testes [134]. Mutations in these genes can lead to fibrinogen deficiencies and disorders of coagulation, and therefore are considered as an origin of coagulopathies [135]. For instance, mutations in the *FGG* gene cause an abnormal conformation of the molecule, leading to FG aggregation within the ER (Figure 3B) [133], which results in a condition known as HHHS, a rare autosomal dominant genetic disorder characterized by low levels of immunoreactive FG in the blood (<150 mg/dL, normal levels should be between 200–400 mg/dL), as well as aggregation of FG in the form of fibrils within the hepatocellular ER [134]. Therefore, similar to AATD, HHHS predisposes to progressive liver disease [133] (Figure 3C).

### 4.2. FG Aggregation in the Cell

The molecular mechanisms of FG aggregation in the ER are currently not well understood. However, previous investigations indicate that mutations in the region located between residues 310 and 401 appear to be required for this process [138]. Indeed, based on the similarities between the structures of the γ-module and those of serine protease inhibitors of serpins, one hypothesis proposes that this region might play functional roles through its insertion or deletion from the central β-sheet [134,139]. The main β-sheet of FG constitutes the support of a mobile reactive center loop; this area is used as bait for its target protease. In serpins, after protease cleavage, the reactive loop is incorporated in the center of the five-stranded β-sheet and compacted against the serpin, inhibiting the enzyme by modifying its active site [140]. Thus, the mechanism of β-strand removal in FG aggregation would allow the C-terminal end of the chain to extend from the FG molecule, allowing the protein to bind to potential interactors, promoting the aggregation process (Figure 2B) [131,134,135]. 

On the other hand, the mutations present in the γ-chain cause conformational changes in the globular domain region involved in the previously described interaction, which impairs the formation of the D-dimer. Thus, all monomeric γ-FG transcripts have an abnormal exposure of hydrophobic regions that can interact with apolipoprotein B and lipids, inducing their intracellular aggregation [141]. Interestingly, the aggregation observed in the ER can lead to unbalanced homeostasis and is considered the central event in the pathogenesis of HHHS [135]. Furthermore, inclusions of FG have been classified into 3 types (Table 1). Type I FG inclusions are related to FG mutations and coagulopathies. On the other hand, type II and III mutations are related to liver diseases not associated with FG deficiency but have been found in some recent HHHS biopsies [134,141]. In this regard, HHHS, as previously described for PD and AATD, is a misfolding-associated disease caused by mutations that result in ER protein storage leading to severe organ-specific damage (Figure 2).

### 4.3. Physiological Response to FG Aggregation: Autophagy and Proteosomes

HHHS represents an ultra-rare disorder with a prevalence of <2 cases per 100,000 people [134]. Under normal conditions, hepatic cells such as HepG2 cells, degrade overexpressed fibrinogen chains through proteosomes, γ chains being degraded much slower (more than three hours) than Aα and Bβ chains (one and a half hours) [142]. Given the longer half-life of free γ chains and the lower rate of transport from the ER to proteasomes than the other fibrinogen chains, there is speculation that the unequal rates of degradation may contribute to the accumulation of surplus γ chains in hepatocytes [143]. Notwithstanding, although many molecular-genetic studies have been conducted, the precise mechanism of mutant FG degradation remains unclear (Figure 3D). 

In this regard, Kruse and colleagues [144] showed that FG degradation was associated with both an ER-associated clearance and autophagy in yeast. Their proposed model for the clearance of aberrant FG involves three quality control processes: a first approach through ERAD, autophagy, and, in the case of saturation due to elevated concentrations, the removal of the soluble misfolded proteins via the biosynthetic pathway of the vacuole. Additionally, Le Fourn and colleagues demonstrated that FG Aα-γ assembly intermediates were selectively cleared by autophagy in human liver carcinoma cells [145]. Finally, Puls and colleagues observed that the carbamazepine, a well-tolerated anticonvulsive treatment known to enhance autophagy, was capable of normalizing alanine aminotransferase levels in HHHS patients (which are usually increased with respect to the upper normal level) [146]. These data point to autophagy as the main pathway responsible for intracellular FG clearance; nonetheless, further research must be carried out to fully confirm this asseveration. 

## 5. Endoplasmic Reticulum Stress and Unfolded Protein Response

### 5.1. The ER and the ER Stress

The ER is the organelle responsible for the synthesis and secretion of membrane proteins, which involves regulation of folding and post-translational modifications. The coordination of the ER in the protein folding process involves a translational phase and a post-translational phase in which a newly synthesized protein in the ribosomes undergoes a series of modifications in interaction with molecular chaperones and folding enzymes (collectively called ER chaperones) that assist in correct folding and exit of proteins from the ER prior to their subsequent transport into the Golgi apparatus [147,148]. The main modifications that occur during folding are cleavage of the signal peptide by the signal sequence peptidase complex, glycosylation, disulfide bond formation, pro-isomerization, and oligomerization. All these modifications are associated with translational and post- translational phases, except for oligomerization, which involves only post-translational modifications [148].

Unfolded proteins are retained in the ER, retrotranslocated to the cytoplasm, and degraded by the proteasome. Nonetheless, when protein synthesis exceeds the folding capacity of the ER, either because of a malfunction of the system due to cellular alterations that affect folding efficiency or because of mutations in proteins that affect their structure or concentration, unfolded proteins accumulate in the ER [149,150]. Misfolding leads to the exposure of hydrophobic amino acid residues that are located inside the protein, which prone the aggregation of other misfolded proteins, resulting in the formation of inclusions [147]. As mentioned above for α-syn, AAT, and FG, these aggregates are so toxic that they cause conformational diseases, such as neurodegenerative disorders and liver conditions, mainly due to the alterations following misfolding and protein accumulation, such as decreased calcium levels, increased oxidative stress and glycosylation dysfunction [151].

This stressful situation known as ER stress activates a series of self-defense mechanisms collectively referred as the Unfolded Protein Response (UPR), which vary depending on the cell type and proteins involved [152]. In this section of the review, we describe the basics of the UPR and compare the specific mechanisms observed for α-syn, AAT, and FG, to emphasize the importance of addressing protein aggregation and improving the defensive ER responses as potential common clinical treatments for PD, AATD, and HHHS.

### 5.2. The Unfolded Protein Response

The UPR of eukaryotic cells consists of a network of signal transduction pathways that activate gene transcription, mRNA translation, and protein modifications to reduce the amount of unfolded or misfolded proteins to restore ER homeostasis [153,154,155]. In mammals, the UPR pathways can be separated into three main signaling cascades initiated by the following ER transmembrane protein sensors: protein kinase R-like endoplasmic reticulum kinase (PERK), inositol-requiring kinase 1 α-subunit (IRE1α), and activating transcription factor 6 (ATF6) [156]. These signal transducer molecules possess luminal ER domains that sense unfolded proteins and cytosolic regions that generate signals to protect cells from ER stress under pathological conditions [157]. The first reaction to ER stress is carried out by PERK, a kinase that phosphorylates the eukaryotic initiation factor 2 α-subunit (eIF2α), leading to the attenuation of protein synthesis preventing the influx of newly synthesized proteins into the ER [156]. On the other hand, signaling through IRE1α, a type 1 ER transmembrane protein kinase, involves its oligomerization and autophosphorylation under ER stress [158], which elicits its ability to excise a portion from the mRNA encoding the transcription factor X-box-binding protein 1 (XBP1) that hence expresses an active XBP1 transcription factor (XBP1s) that upregulates genes involved in the degradation of misfolded proteins [159]. Finally, ATF6 is cleaved on ER stress into a fragment known as ATF6p50, that in conjunction with XPB1s, translocates to the nucleus and regulates transcription of genes encoding ER chaperones and enzymes responsible for the degradation of misfolded proteins [160]. 

Overall, the UPR represents a combination of signaling pathways that maintain ER proteostasis and sustain cell function under ER stress by adjusting the ER folding capacity. Nonetheless, when the capacity of UPR to maintain proteostasis is overwhelmed, cells activate the control of cell death by apoptosis [157]. Today, the mechanisms underlying the transition from cell survival to cell death under ER stress remain largely unknown [153]. Furthermore, different UPR pathways may be activated for unique pathophysiological processes in specific cell and disease states. Because of this, we will proceed to survey what we know so far regarding the defensive mechanisms of ER stress and the UPR response in these conditions. 

### 5.3. ER Stress and UPR in PD

So far, the involvement of the UPR in PD has been demonstrated in the toxin-induced PD models of 6-hydroxydopamine (6-OHDA), methyl-4-phenyl-1,2,3,6-tetrahydropyridine (MPTP), and rotenone [161]. Hoozemans and colleagues were the first to report the relationship between the activation of the UPR system and PD [162]. They observed increased levels of phospho-R-like endoplasmic reticulum kinase (phospho-PERK) and phospho-inositol-requiring kinase 1 α-subunit (phosphor-eIF2α) proteins, as well as 78-kDA glucose-regulated protein (GRP78/BiP), activating transcription factor 4 (ATF4), and the transcription factor CHOP in SNpc from post mortem samples of patients with PD compared with controls [163,164]. Nevertheless, activation of the UPR system in PD disease was confirmed with the accumulation of the protein-disulfide isomerase protein (PDIp), a member of the disulfide isomerase family associated with disulfide bond formation, reduction, or isomerization of nascent proteins [165,166], which constitutes an adaptive and neuroprotective response against ER stress [167].

Regarding the etiology of UPR activation in PD, α-syn overexpression represents an important factor as it correlates with chronic activation of multiple pathways of the UPR system and ER stress-mediated apoptosis. For instance, in yeast models, it has been shown that the UPR system, although remaining active under basal conditions, can be overactivated by α-syn overexpression [168]. This response leads to alterations in cellular homeostasis, reflecting in an increase in ER stress that can conduct to cell death. Similarly, dopaminergic neuron cultures with triplication of the *SNCA* gene have been observed to exhibit increased ER stress due to the high levels of α-syn [169]. This process leads to a dysfunction of homeostasis, mainly in calcium concentrations, which activates the apoptotic cell death pathway [170]. Particularly, SH-SY5Y cells treated with oligomeric (pathogenic), but not monomeric, α-syn exhibited increased XBP1 splicing: these data indicate that there is an oligomer-specific activation of α-syn oligomers in the IRE1-XBP1 signaling pathway. Furthermore, several studies have reported that mutant, phosphorylated and oligomeric α-syn activates all three branches of the UPR, triggers ER stress-induced apoptosis, and promotes the induction of autophagy [171,172,173]. Finally, the most direct relationship between ER stress activation and α-syn neuropathology possibly arises from alterations in Beclin-1 and LC3 expression, which is mediated by ER stress activation through the effects of α-syn aggregation that together are implicated in autophagy induction [174]. However, despite being crucial against α-syn aggregation, ER stress and UPR response are also susceptible to impairment due to α-syn mutations that trigger its fibrilization. Thus, the A30P α-syn mutation affects mRNA levels of UPR genes in vitro and in vivo and induces Golgi fragmentation in LUHMES (Lund Human Mesencephalic) cells [175], whereas overexpression of α-syn by the A53T mutation increases BiP and phospho-eIF2α levels [176]. Likewise, inhibition of the RAB pathway by mutated α-syn is implicated in ER stress activation and impairment of the autophagy–lysosomal pathway [177]. However, further studies combining the determination of the mechanisms involved between ER/UPR and autophagy are required to explore the pathological mechanisms of PD to develop intervention strategies.

Nonetheless, all this evidence does not allow us to determine whether α-syn neurotoxicity is the cause or the consequence of UPR failure, nor does it allow us to know which is the main trigger of PD pathogenesis. However, recent work by Colla and colleagues in A53T transgenic mice indicates that accumulation of toxic α-syn species in the ER is responsible for UPR activation [178] and that detection of α-syn oligomers associated with this organelle precedes the stress response [179]. This evidence suggests that UPR activation is a consequence of α-syn accumulation in the PD. Yet, further research is still needed. In parallel, there is an increase in mitochondrial stress, which triggers ER stress, affecting the UPR function upon α-syn misfolding and aggregation, resulting in PD neurodegeneration [180].

### 5.4. ER Stress and UPR in AATD

The effect of Z-AAT expression on ER stress has mostly been studied through cell culture models, human monocytes and airway epithelial cells, and human and animal liver biopsies [181,182]. Nonetheless, although there are some AATD studies related to ER stress, nowadays it remains unclear how Z-AAT polymers activate the UPR [183]. 

It has been proven that UPR can be activated in response to overexpression of Z-AAT in HEK293, HepG2, and 16HBE14o-cells [184,185], however, these pathways do not appear to be activated in inducible models of AATD liver disease or in liver cells in vivo, as several studies have failed to detect activation of the UPR in cell culture and animal liver models of AATD [127,186]. It has therefore been speculated that the absence of UPR signaling allows the survival of cells that have accumulated high levels of Z-AAT. Likewise, the activation of UPR in human peripheral blood monocytes [187], but not in HeLa cells [186] nor rat liver [188], could be explained by the UPR needing secondary stress to be activated. In this regard, Lawless and colleagues observed that in CHO cells, UPR was not activated when Z-AAT polymers were expressed alone, but when they added thapsigargin (an ER stressor) or heat stress [189]. Ordóñez and colleagues [190] also supported the theory of the second stressor by observing that Z-AAT only activated the ER overload response, whereas truncated AAT mutants only activated the UPR. This is significant considering that these two pathways usually occur together. Their data revealed that Z-AAT accumulation into inclusion bodies produces a loss of the normal tubule ER network, forming a vesiculated ER and leading to impairment of luminal protein mobility. On the contrary, truncated AAT polymers cause classical ER stress (UPR) and are efficiently degraded by the proteasome, showing a different ultrastructural change characterized by gross expansion of ER cisternae. Furthermore, the increased ER stress sensitivity observed following Z-AAT expression correlates with marked changes in the biophysical characteristics of the ER. When cells experience ER overload, misfolded proteins are uncapable to diffuse freely: this decreases their accessibility to the folding and transportation mechanisms. By contrast, in reticular and highly interconnected ER cells, chaperones can diffuse to misfolded proteins’ sites. Consequently, Hidvegi and colleagues proposed a model in which decreased mobility or availability of ER chaperones sensitizes the cell to subsequent activation of the UPR [186].

In addition to the above, it has emerged that AATD may also be associated with aberrant immune cell function [187]. Carroll and colleagues observed UPR activation in monocytes from patients with AATD and linked this phenomenon to an altered inflammatory response. In this work, they observed that most genes involved in the UPR increased in monocytes from ZZ patients compared to MM individuals. Furthermore, this gene expression can be induced in MM monocytes by adding thapsigargin, linking the observed ZZ monocyte changes to ER stress. Thus, our current understanding of the mechanisms regulating Z-AAT-related lung and liver disease should now be expanded to include a role for exaggerated inflammatory responses by circulating blood cells. 

Nonetheless, the proteasomal pathway does not fully account for disposal of all Z-AAT, autophagy being necessary as it has been mentioned before [191]. Thus, the current dogma regarding Z-AAT elimination involves two mechanisms, the ubiquitin–proteasome system activated by the UPR, and autophagy. The first deals with removal of soluble Z-AAT that accumulates in the ER whilst autophagy degrades polymerized and aggregated forms of Z-AAT that become abundant during the acute phase response when expression of AAT is induced. Indeed, when the balance between the misfolded protein load in the ER and the ability of the cell to correct ER homeostasis cannot be restored, cell death via apoptosis remains the ultimate mechanism to avoid further damage into other cells [192]. 

### 5.5. ER Stress and UPR in HHHS

Assembly of all six chains that constitute the FG molecule occurs within the hepatocyte ER [193,194]. Normally, individual unassembled FG chains are retained within the ER and ultimately degraded in a proteasome-dependent manner [143], as previously described. Likewise, the soluble form of misfolded FG can be degraded by the proteasome through ER-related protein degradation; nonetheless, in the circumstance that these mechanisms are inhibited, autophagy is significantly activated [195]. Conversely, the data of Puls and colleagues [146] continue to identify autophagy as the main degradation mechanism for aggregated FG, as they provide evidence for feasibility of therapeutic exploitation of pharmacological enhancement of autophagy in FG liver storage diseases. Nowadays, to our knowledge, there are no studies linking any UPR activation pathway to FG aggregation within ER, nor any ER stress response associated. However, having in consideration the similarities between the mechanisms by which accumulated mutant AAT and FG mediate cellular toxicity leading to hepatic storage diseases, there could be a potential UPR-mediated mechanism of protein degradation like that observed for Z-AAT in AATD. Further research must be carried out addressing if this is the case.

## 6. Hallmark Findings Comparison

**Figure 4:**
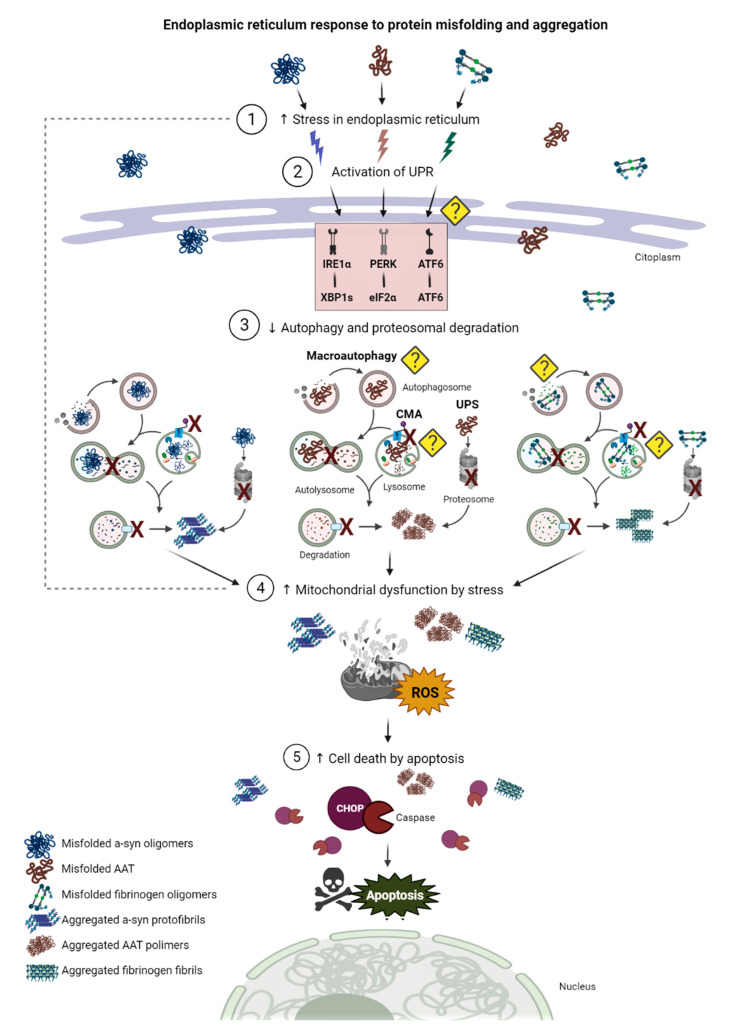
ER response after misfolding and aggregation of α-syn, AAT, and FG. (**1**) The ER increases its stress levels upon protein misfolding and aggregation produced by the dysfunction of homeostasis in the cellular milieu [208,209]. (**2**) The UPR system is activated by the following stress sensors IRE1-α, PERK, and ATG-6, which in turn activate the transcription factors XBP1 and eIF2α involved in the regulation of the ER stress response and autophagy pathway-dependent degradation [150,208,209]; for the case of FG, there are no studies linking UPR with FG misfolding and aggregation, however, considering the similarities of FG with AAT in terms of cellular toxicity, it is likely that similar defensive processes take place [210,211]. (**3**) The main degradation systems: the autophagic pathway and the ubiquitin–proteosome system (UPS) deteriorate their function in the face of increased misfolded proteins [62,130,143]. This prevents the correct degradation of proteins, causing an increase in their aggregation. Within the autophagic pathway, in the case of α-syn, macroautophagy and CMA are known to mediate the degradation of this protein upon misfolding [69]. In contrast, for the case of AAT and FG, the types of autophagy involved in their degradation have not yet been elucidated [114,116,195]. (**4**) In parallel, there is an increase in mitochondrial stress, which affects its UPR function upon α-syn (dotted line) [176], AAT [181], and FG [212] misfolding, leading to dysfunction of this organelle. (**5**) Finally, dysfunction of the above pathways leads to activation of the transcription factor CHOP (C/EBP Homologous Protein) that directly or indirectly potentiates the activity of caspases, culminating in cell death by apoptosis [143,184,211,213].

The main comparison between the pathologies reviewed is summarized in Figure 4 and Table 1. Cell damage induction differs between pathologies, both in the type of aggregates and in the pathophysiological mechanisms. Polymers of α-syn can be found intracellularly and extracellularly, indicating that they can be translocated to other neurons [196,197]. However, accumulation and aggregation of α-syn take place mainly in the cytoplasm in the form of LBs [198], being able to interact with many organelles [199], especially mitochondria [200], and ER [201], causing damage that leads to cell death [51,202]. In contrast, mutated AAT misfolds and aggregates only in the ER, and its transport to the Golgi apparatus is blocked [104,203], leading to proteotoxicity liver injury due to activation of the ER overload response and upregulation of inflammation-related genes, including NFκB signaling [100,200,201], as observed with other proteins accumulated in ER [204]. Likewise, pathological events due to Z-AAT accumulation effects are not limited to the ER, as increased autophagy, mitochondrial injury, cytochrome c release, as well as caspase 3 activation, have been observed in different models of AATD [205,206], as a cellular response that may result in apoptosis [206]. Interestingly, caspase 3 activation also occurs in neurons cultured with α-syn-conditioned medium [196], indicating that there are common mechanisms (e.g., caspase activation, mitochondrial impairment) in different pathologies of protein accumulation ending in apoptosis [103,206]. Finally, given that the hepatic conditions of AATD and HHHS share certain similarities [108], it is likely that the ER-stress response produced by FG aggregation resembles that observed in AAT, however, the specific pathway(s) and its products are yet to be elucidated [134]. Nonetheless, the inclusions observed in HHHS are different from those generated by α-syn or Z-AAT: type I present polygonal shapes, type II have ground-glass appearances, and type III are eosinophilic globules with granular structures in their periphery [108,207].

Regarding the cell physiological response to protein accumulation, certain pathways are shared upon protein accumulation. Although the activation pathways following different stimuli elicited by α-syn, AAT, and FG vary due to their intrinsic properties, all three diseases (PD, AATD, and HHHS) share initial chaperone responses as the first line of defense against misfolding and accumulation of the proteins involved [214]. If this does not lead to proper folding, the protein is ultimately degraded either by specific ERAD mechanisms [215] or by presumably constitutive turnover of the ER by autophagy [216]. The cellular preference for one or the other varies according to the type of protein accumulated and the cell line involved. For instance, in hepatocytes, AAT accumulation is mainly resolved with autophagy, as previously described. However, clearance by proteosomes, especially through ERAD pathways, exerts some importance in the removal of misfolded AAT. Curiously, genes involved in this mechanism also activate autophagy when ubiquitinated proteins are not degraded, suggesting a direct relationship between the two mechanisms. The above has also been observed for FG, where autophagy has been identified as the main mechanism of protein clearance, acting in response to a saturation of the proteolytic systems of the liver cell. Nonetheless, for the case of FG there is insufficient information to recognize a potential relationship between the two responses. In contrast, degradation of α-syn in dopaminergic neurons follows two specific autophagy pathways: macroautophagy and CMA, where an important relationship is observed between the accumulation of α-syn and the deterioration of the autophagic response, since both the malfunctioning of the ALP leads to the aggregation of α-syn and this aggregation reduces the effectiveness of the pathway, which ultimately leads to cell death and neurodegeneration.

Finally, if protein-folding homeostasis is compromised and misfolded proteins accumulate in the ER, the cell experiences ER stress [217], a surveillance system that can trigger the UPR, which either enables the cell to recover proteostasis, or if this process fails, to bring about cell death [213]. As described in the previous section, both ER stress and UPR are dependent on pathophysiological conditions. For example, while overexpression of α-syn and Z-AAT on in vitro models has been observed to activate the UPR, it remains unclear if FG aggregation in hepatocytes leads to UPR. Nonetheless, the lack of literature in this regard still leaves open the possibility of potential ER responses to their accumulation given their similarity to the pathophysiological processes of AAT accumulation. What remains clear is the need for further deepening in our knowledge regarding the UPR response to α-syn, and AAT and FG accumulation in dopaminergic and hepatic cells, respectively. This is because an unsatisfactory response by the proteostasis systems undoubtedly leads, for all three proteins, to apoptotic cell death, which results in the neurodegeneration and liver disorders observed. However, it is still not clear how the multiple pathological events of these diseases, mainly protein aggregation in the ER, are related and/or lead to cell death by apoptosis. Particularly for the case of PD, the few studies in this regard focus on the effects of α-syn on mitochondrial dysfunction that triggers apoptosis. Consistent with this, mechanisms of α-syn interaction with the mitochondrial membrane, deletions in mitochondrial DNA, and mitochondrial dysfunction in SNpc dopaminergic neurons have been identified [210,211]. In addition, previous studies have observed that dysregulation of autophagy leads to the accumulation of α-syn, as also observed for Z-AAT and FG, and, ultimately, to cell death by apoptosis. Autophagy stimulates apoptosis mainly by an increase in proapoptotic proteins. Nonetheless, it is not yet known whether autophagy acts as a mechanism to prevent or induce apoptosis [218]. On the other hand, recalling the role of the ER in Ca^2+^ regulation and its role in PD by increasing ER stress and activating the UPR system, Ca^2+^ is another molecular component strongly involved in the regulation of cell death in PD [219]. In this regard, Ca^2+^ may also have an indirect relationship with apoptosis in hepatic cells in AATD, as it has been reported that perturbations of cellular calcium blocks exit of AAT from the ER, leading to its misfolding [220,221]. As mentioned before, if proteostasis is not recovered, hepatocytes turn to apoptosis.

## 7. Clinical Perspectives

### 7.1. Proteolytic Pathways Induction as Potential Treatment for α-Syn Aggregation in PD

Currently, the main focus of research on α-syn and PD is to understand the mechanisms involved in the formation of soluble α-syn oligomers and their specific conformations [50], as well as the defensive mechanisms that take place upon aggregation. The discussion in the previous sections confirms that the accumulation of α-syn in SNpc dopaminergic neurons triggers an ER stress process that activates the UPR system and may lead to autophagy: if these defensive responses are not sufficient, apoptosis-mediated cell death processes take place. Therefore, clinical perspectives regarding PD should focus on three main approaches: (i) the pathogenic evolution of α-syn, (ii) ER stress and UPR mechanisms, and (iii) autophagy. The main clinical perspectives for α-syn aggregation in PD are summarized in Table 2.

With respect to α-syn oligomerization, research is still in its beginning phase. Understanding the properties of α-syn oligomers in the early stages of the disease will allow the development of diagnostic techniques and prevent the development of toxic effects towards neurodegeneration. In this regard, inhibition of aggregation could represent an immediate solution: it has been evaluated in transgenic mice that treatment with salubrinal, an ER stress compound, reduces the accumulation of α-syn oligomers in the ER, further confirming a link between α-syn and ER stress [179]. Furthermore, future research should focus on the development of drugs with high affinity and selectivity for the toxic conformation of α-syn, as well as strategies that show the degree of disease progression and the development of drugs for treatment at different stages.

Certainly, if aggregation is not inhibited, ER stress and UPR must be activated and enhanced in case of impairment. In this respect, ER stress-related molecular compounds, mitaramycin and methoxyflavones, have been shown to reduce ER stress-induced neurotoxicity in vitro [222], and activate the UPR system [223], respectively. Furthermore, several studies suggest that regulators of the UPR pathway, such as PERK and XBP1s, could play a role in neuroprotection against PD. On the other hand, transgenic mice deficient in XBP1, show an increase in the accumulation of misfolded proteins such as a-syn, attributed to a decrease in the functioning of degradation mechanisms, which activate the autophagic pathway [224]; likewise, deregulation of FOXO1, and other genes involved in autophagy, leads to the inhibition of XBP1 compromising cell viability [225]. Moreover, genetic deletion of ATF6 potentiates susceptibility to neurotoxins in PD mouse models [226,227], while overexpression of Bip/Grp78 exerts neuroprotective effects [228].

Finally, to avoid cell death, autophagy is necessary, hence the importance of autophagy-enhancing approaches. In line with this idea, in vitro activation of macroautophagy by lentiviral expression of the inducer Beclin-1 decreased α-syn immunoreactivity and recovered neuronal integrity [174]. Similarly, induction of macroautophagy by administration of rapamycin, trehalose, or overexpression of ATG-7, decreases neurodegeneration and behavioral alterations induced by α-syn aggregation [229,230]; nonetheless, rapamycin, although protective against apoptosis [231], is known to exacerbate the neurotoxic effect of monomeric α-syn overexpression [63] and α-syn aggregation induced by p internalization of pre-formed fibrils (PFF) into α-syn expressing HEK293 cells or primary cultured neurons [58], so treatment should take place before α-syn aggregates are formed. Furthermore, activation of the CMA pathway by Lamp2a overexpression in vivo decreases α-syn WT-mediated neurotoxicity, increases the survival of SNpc dopaminergic neurons and the functionality of their striatal terminals [69,232]; however, further studies are required to understand the effects of CMA regulation with the purpose of developing therapeutic goals. Finally, there is a close relationship between the GCase and α-syn aggregation, suggesting that current research should give a therapeutic approach associated with the effects of mutant GCase or WT on lysosomes, which could prevent or decrease the formation of toxic α-syn species. In a first approach, overexpression of WT GCase decreases soluble α-syn levels in α-syn A53T transgenic mice [233].

**Table 2:** Targets for clinical strategies against α-syn neurotoxicity in PD.

Parkinson’s Disease				
Target	Strategy	Results *	Conclusions	Ref.
ER stress	Transgenic mice over-expressing WT or mutant (A53T and A30P) α-syn treated with Salubrinal	↓ α-syn oligomer ↓ ER stress	α -syn oligomers cause neurodegeneration by chronic ER stress response in vivo	[171]
ER stress	Mithramycin (MTM) administration in organotypic hippocampal slice cultures	↓ ER stress-induced neurotoxicity ↓ Cell death by CHOP inhibition	MTM is a protective agent against ER stress neuronal death in vitro	[214]
ER stress	Tangeretin administration in mice injected with tunicamycin	↑ Expression of GRP78 in SNpc ↓ Cell death induced by MPTP	Tangeretin regulates ER stress-related to PD	[223]
ER stress and UPR	Genetic deletion of ATF6α in transgenic mice treated with MPTP	↓ TH levels and ↓ Number of dopaminergic neurons in SNpc	ATF6α exerts neuroprotection of dopaminergic neurons from MPTP toxicity in vivo	[218]
ER stress and UPR	Mouse model with deletion of ATF6α gene and injection of MPTP and probenecid (MPTP/P)	↓ GRP78 ↑ Neuronal degeneration ↑ Ubiquitin accumulation ↓ Astroglial activation ↓ BDNF ↓ Anti-oxidative genes ↓ CHOP	UPR is activated in a model of chronic MPTP/P injection causing neurodegeneration	[219]
ER stress and UPR	Administration of tangeretin into mice with deletion of ATF6α and MPTP/P	↑ UPR-target genes ↑ Dopaminergic neuronal survival ↑ Astrocyte survival	UPR contributes to the survival of dopaminergic neurons in SNpc	[219]
ER stress and UPR	Overexpression of chaperones GRP78/BiP in α-syn rat model of PD	↓ α-syn neurotoxicity ↓ Apoptosis in TH neurons of SNpc ↑ Levels of striatal dopamine release	The GRP78/BiP plays a neuroprotective role in α-syn neurodegeneration	[220]
Macroautophagy	Overexpression of α-syn in cell cultures (SKNSH, HeLa and HEK293 lines)	↑ p62 and ↓ LC3-II ↓ RAB1 homeostasis ↓ Omegasome formation Mislocalization of ATG-9	Rab1a, α-syn, and ATG-9 regulate the formation of Omegasome	[60]
Autophagy–lysosome system	Overexpression of α-syn by lentivirus transduction and co-expression of Beclin-1 in a neuronal cell line	↓ Accumulation of α-syn ↓ Neuritic alterations ↑ Effects of Beclin-1 by Rapamycin ↑ Lysosomal activation ↓ Synaptic and dendritic pathology ↓ Alterations in autophagy pathway	Beclin-1 decreases neuronal pathology of α-syn by inducing autophagy in vitro	[174]
Macroautophagy	Induction of macroautophagy by administration of trehalose in A53T α-syn transgenic rats	↓ α-syn accumulation and aggregation in SNpc ↓ α-syn deficits in motor asymmetry ↑ Survival of dopaminergic neurons ↑ Dopamine turnover	Induction of macroutophagy prevents/ reverse α-syn aggregation in models of PD	[230]
CMA	Overexpression of LAMP2A in SH-SY5Y cells, rat cortical neurons in vitro, and SNpc neurons in vivo	↓ α-syn neurotoxicity ↑ Survival of SNpc dopaminergic neurons ↑ Functionality of dopaminergic striatal terminals	Induction of CMA provide a novel therapeutic strategy for treatment of PD	[232]
Autophagy–lysosome system	Overexpressing of GCase in A53T α-syn transgenic mice	↓ Soluble α-syn levels	GCase represents a potential therapeutic strategy for PD	[233]

* Arrows indicate increase (↑) or decrease (↓) of specific result.

Nonetheless, although autophagy plays a crucial role in the degradation of toxic aggregation-prone proteins as well as in the maintenance of cellular homeostasis during various stress conditions, there is evidence that increased levels of autophagy can be detrimental and induce programmed cell death [234,235], as it has been observed in a familial ALS murine model upon autophagy induction [236]. In neurons, autophagic cell death (ACD) has been linked to an elevated number of cytoplasmic autophagosomes, which are believed to lead to excessive degradation of cellular components [237]. This phenomenon has been observed in brains of PD patients [238,239]; however, it remains unclear whether these accumulations of autophagosomes may represent a failed rescue response to lethal stress, rather than a direct lethal mechanism per se [235,240]. In this regard, we believe that the beneficial or counterproductive role of autophagy in the face of PD neurodegeneration likely depends on: (1) the degree of neurodegeneration by α-syn aggregates in cells and the overall autophagic load; (2) the neuronal capacity to increase autophagic flux; (3) the redox state, mitochondrial function, and signaling of cell death pathways; (4) the ability to clear protein aggregates; and (5) the timing of the involvement of both pro- and anti-autophagic processes. Thus, if autophagy induction can lead to ACD (at least in neurons), the design of new autophagy-based proteolytic techniques should consider this risk; similarly, it should be elucidated whether these mechanisms of cell death can also occur in other cell types and pathologies, such as ERSDs. It is important to mention that the development of therapies involving a decrease of α-syn inclusions cannot be tested in tissue biopsies, but only in post mortem samples from patients diagnosed with PD. Such tissues must be subjected to different stains with specific markers that allow detection of the phosphorylated protein, both in its amyloid structure and in its aggregated form [233]. Furthermore, it is critical to mention that recent studies by Lau et al. [241] indicate that the strain of α-syn involved in aggregation has an impact on the type of aggregates at the cellular level, as well as in the cell type in which they occur, contributing to the clinical variety of phenotypes of synucleinopathies. In this regard, Ferreira et al. [242] identified a novel α-syn strain (α-Syn/p25α) induced by multiple system atrophy-associated oligodendroglial p25α protein. This strain was observed to have a faster and more aggressive phenotype than recombinant α-syn in terms of a higher α-syn aggregate load and enhanced neurodegenerative potential. These new findings reflect the importance of studying the development of α-syn strains when developing new research to deepen our knowledge of these diseases, which will serve as a basis for more efficient therapeutic strategies. 

### 7.2. Proteolytic Pathways Induction as Potential Treatment for AAT Aggregation in AATD

Given that to date liver transplantation remains the only therapeutic option for patients with AATD [243], the development of modern therapeutic approaches that address the pathological conditions underlying the disease to prevent its progression is crucial. Similarly, it is necessary to deepen the understanding of the defensive mechanisms that the cell performs against aggregation. As mentioned in the previous sections, the ER is the main location of Z-AAT accumulation in hepatocytes when the ERAD pathways fail to clear mutant and misfolded AAT proteins, leading to ER stress, the activation of UPR mechanisms, and, if necessary, autophagy. Thereby, clinical perspectives regarding AATD must focus on the three approaches stipulated for PD: (i) the pathogenic evolution of Z-AAT, (ii) ER stress and UPR mechanisms, and (iii) autophagy. The main clinical perspectives for AAT aggregation in AATD are summarized in Table 3.

With respect to AAT polymerization, altering misfolding can lead to beneficial effects [244]. Polymerization may be prevented by the development of small peptide inhibitors, as shown by an in vitro work in which Z-AAT aggregation was prevented by a 6-mer peptide that selectively binds to the pathogenic serpin conformation [245]. On the other hand, chaperons are the most important protein molecules for the correct folding and localization of proteins. Some chemical compounds, like glycerol, trimethylamine N-oxide, and 4-phenylbutyric acid, are known to have such activity [246]. In particular, 4-phenylbutyric acid has been shown to mediate an increase in Z-AAT secretion in cell culture and murine models [247]. Likewise, trimethylamine N-oxide has been shown to stabilize native AAT [248]. However, it is unclear whether long-term administration of these compounds will reduce the AAT load retained in the ER of liver cells. The above represents a potential approach to AATD in the early stages of AAT misfolding and aggregation. 

As mentioned above, there is evidence linking AAT aggregation and ER stress; however, activation of the UPR is inconsistent [249]. Recently, Z-AAT has been shown to interact with IRE1α and induce the ATF6 binding responsive reporter activity [250]; in this regard, induction of the ATF6 pathway has been shown to attenuate Z-AAT accumulation and mitochondrial damage in liver cells through promoting ERAD [251]. 

**Table 3:** Targets for clinical strategies against AAT in AATD.

α-1-Antitrypsin Deficiency				
Target	Strategy	Results *	Conclusions	Ref.
Block polymerization of Z-AAT	Administration of 6-Mer reactive loop peptide (FLEAIG)	↓ Polymerization of Z- AAT	Small molecule inhibitors can be used to treat Z-AAT deficiency.	[245]
ER stress and UPR	Administration of modulators of UPR: Sarcosine, Betaine, Hydroxyectoine and Ectoine in ER-stress induced by Tunicamycin	↑ Restoration of homeostasis ↓ Levels of GRP78 and ATF-4	Modulators of UPR mitigate the pathophysiological state of ER-stress.	[246]
Reverse misfolding of AAT	Administration of chemical chaperone: 4-phenylbutyric acid (PBA) in cell culture system and Z-AAT mice	↓ Z-AAT secretion levels in cell culture and murine models	PBA is an important treatment of target organ injury in AAT deficiency	[247]
Polymerization of Z-AAT	Administration of trimethylamine N-oxide (TMAO)	↓ Conversion of the native state to a polymerogenic intermediate	TMAO control the conformational transitions of folded AAT	[248]
Autophagy	Administration of autophagy enhancing drug carbamazepine (CBZ) in HeLa cell line HTO/Z and ATG-5–deficient cell line	↓ Levels of ATZ insoluble and soluble fractions ↑ Autophagic flux by LC3-I and LC3-II ↓ Levels of soluble and insoluble ATZ in ATG-5 deficient line	CBZ is efficient in AAT deficiency as autophagy enhancer.	[110]
Autophagy	Activation of ATF6 by expression of spliced ATF6 (1–373 exons)	↑ ER-associated degradation of Z-AAT ↓ Hepatocyte loss	ATF6 pathway limits Z-AAT cell toxicity	[251]
Autophagy	Cell lines (mouse embryonic fibroblast) with deletion in ATG-5 gene	↓ Degradation of Z-AAT ↑ Z-AAT inclusions	Autophagic degradation prevent toxic accumulation of Z-AAT.	[235]
Autophagy	Effect of rapamycin on mouse model of Z-AAT	↑ Autophagic activity by number of vacuoles ↓ Intrahepatic accumulation of Z-AAT ↓ Caspase 12 levels ↓ Hepatic fibrosis	Rapamycin reduces polymerized Z-AAT and progression of liver injury.	[236]
Autophagy	Liver-directed gene transfer of transcription factor EB (TFEB) in a mouse model of SERPINA1 deficiency.	↓ Expression of SERPINA1 monomer ↑ Degradation of SERPINA1 polymer by autolysosomes ↓ Apoptosis and fibrosis 236	TFEB gene transfer is a novel strategy for liver disease in SERPINA1 deficiency and prevent accumulation of toxic proteins.	[237]

* Arrows indicate increase (↑) or decrease (↓) of specific result.

Nonetheless, the main perspective in clinical treatments for AATD is clearly the potentiation of autophagy, as Z-AAT accumulation activates a specific autophagic pathway capable of degrading insoluble forms of Z-AAT [252]. Autophagy enhancers have been found to reduce Z-AAT aggregation while preventing the resulting hepatotoxicity. For instance, carbamazepine (CBZ) is an autophagy-enhancing drug with anticonvulsant and mood-stabilizing properties, FDA approved, and widely used in clinical practice. In a 2010 study, the Hidvegi et al. working group demonstrated that CBZ administration increases the degradation of insoluble Z-AAT in in vitro and in vivo models. It does this by increasing autophagic flux, even in autophagically active cells, in addition to increasing proteasomal degradation [116]. Currently, its administration has been subjected to dose response trials for the treatment of severe AATD-mediated liver disease: the results are still inconclusive [253].

Another autophagy enhancer is rapamycin, which has been evaluated in murine models of AATD [254]. In this regard, administration of rapamycin increases autophagic activity and consequently decreases the accumulation of Z-AAT aggregates in the liver. It was also shown to decrease the levels of markers of hepatocellular damage such as caspase-12 and fibrosis. Although these results are promising, there are still no clinical trials demonstrating its effects [243].

Finally, gene therapy has also been proposed as a possible treatment to mediate the aggregation and effects of Z-AAT. In this regard, gene transfer targeting the TFEB gene that regulates lysosomal function and autophagy in transgenic mice significantly reduces Z-AAT levels in the liver. This correlates with increased Z-AAT degradation mediated by increased autophagic flux [255]. Furthermore, TFEB expression decreases the presence of diastase-resistant inclusion bodies, apoptosis, and fibrosis in hepatocytes.

Although significant progress has been made in recent years in identifying the mechanisms and mediators of AATD-mediated liver disease, more questions than answers arise [243]. Thus, studies are required to elucidate and identify personalized approaches for the treatment of AATD.

The storage and accumulation of Z-AAT in hepatocytes is detected histologically by the presence of eosinophilic cytoplasmic inclusions that area visualized by periodic acid-Schiff staining combined with diastase (PAS-D), an enzyme in charge of glycogen degradation. In addition, the identity of these inclusions can be confirmed with antibodies specific for Z-AAT. Therefore, the development of experimental strategies aimed at reducing Z-AAT storage should be confirmed with histological techniques that demonstrate the reduction of inclusions in liver tissue biopsies [233].

### 7.3. Proteolytic Pathways Induction as Potential Treatment for FG Aggregation in HHHS

FG aggregation in HHHS, unlike the other pathological conditions reviewed, remains largely unknown, as do the main mechanisms of ER stress and UPR that take place. In consequence, data on medical management remain scarce. In this regard, clinical perspectives should primarily focus on deepening our current knowledge of the pathophysiological events involved in FG aggregation in hepatocytes thus future treatments could be elucidated once the underlying mechanisms are properly understood. For example, a strong similarity between intrahepatic fibrinogen aggregation and extrahepatic polymerized fibrin has now been discovered. For both there is a lack of hematological manifestations, which represents a challenge for their identification and diagnosis [100]. Therefore, the fibrinogen mutations and alterations causing HHHS require extensive epidemiological studies, as well as the collection of clinical and laboratory work for future research to aid in the diagnosis and treatment of the disease [130,136,138]. On the other hand, it has been identified that upon misfolding and aggregation of FG, a blocking process occurs in the recruitment of the ER and the secretory pathways involved. This discovery will help to study the initial phase of the FG aggregation process and elucidate the structural changes and factors leading to its aggregation [256]. This could guide future research towards interventions capable of decreasing FG storage in HHHS and the development of other coagulopathies [136,253,254].

Nonetheless, despite the knowledge gaps regarding the FG aggregation mechanisms, there are current data pointing to autophagy as the main degradation mechanism involved [146], so the improvement of this proteolytic pathway could represent a solution to HHHS. For instance, CBZ and UDCA treatments have been shown to be beneficial in some cases [146,257]. Of particular interest are the results obtained for the management of patients with CBZ: this drug is a well-tolerated anticonvulsive treatment, known to enhance autophagy, and its efficacy seems to be related to the normalization of ALT levels [146]. The main clinical perspectives for FG aggregation in HHHS are summarized in Table 4.

In conclusion, further research regarding HHHS, and hepatic aggregation of FG remains necessary, as HHHS has been systematically less investigated than AATD. A priority goal must be on the treatment of ESRD with strategies for prevention and hepatotoxic decrease of FG misfolding and aggregation.

### 7.4. Future Research through a Simplified Approach

In addition to proteolysis induction, research on protein accumulation and degradation could follow a different approach, focusing more on normal physiological processes rather than on pathological ones in analogy with models in other fields [258,259]. In this regard, for example, aggregation of Z-AAT and the mutant FG γ-chains could be addressed by studying the behavior of both normal M-AAT in MZ individuals, and the α- and β-chains of FG in HHHS subjects under conditions of clinical stimulation [260], and by studying why extrahepatic cells capable of AAT synthesis do not store the Z-variant in AATD [100]. The first outlook could lead us to understand how we can increase circulating levels of normal protein components capable of preventing intracellular aggregation by binding cleaved Z-AAT and FG γ-chains at the polymerized interface [261], which would accordingly diminish the need to potentiate degradation. This perspective would require the analysis of such molecules by computational modeling, sequence homology, molecular docking, and crystallographic studies, all guided by artificial intelligence, as well as an interaction analysis by assessing the thermodynamic properties of the binding. Conversely, by elucidating the homeostatic mechanisms and intracrine control processes that take place in AAT-synthesizing extrahepatic cells of both M- and Z-AAT phenotypes, we could induce them in the affected liver cells that present the aggregation and, hence, prevent it. 

## 8. Conclusions

The research on α-syn, Z-AAT, and FG misfolding has largely focused on the analysis of the genetic variants of these proteins and the mechanisms by which their aggregation leads to cellular inclusions, which ultimately affect the viability of their respective cell types by interfering with key organelles such as the ER. Certainly, the aggregation process between α-syn and Z-AAT seems to be similar, given the relevant mutations near the reactive center loop that promotes the binding of two or more α-syn or AAT monomers. Likewise, due to its structural similarity with serpins, FG aggregation could have an analogous mechanism. However, its interplay with stress-related defensive mechanisms is yet to be clarified. In contrast, LBs inclusions found in PD differ considerably from those observed in AATD and HHHS, as LBs are constituted by several different proteins and can be found in different organelles and structures of the adult brain, whereas AAT and fibrinogen inclusions are restricted to the ER of hepatocytes and can be initiated during childhood. Additionally, the ER-stress response to α-syn, AAT, and FG aggregation remains to be further elucidated, as actual evidence restricts us to reach a satisfactory conclusion regarding their full pathological characteristics. Nevertheless, as expected, evolutionarily conserved autophagy pathways seem to have parallelisms among α-syn, AAT, and FG. Based on the foregoing, by improving our understanding of the mechanisms involved in α-syn, Z-AAT, and FG aggregation, their effect in the ER, and the defensive cellular responses such as ER stress and UPR, researchers might be capable of developing better procedures to diminish or prevent the misfolding and aggregation process of these proteins, as well as improving the defensive proteolytic pathways, occurring in ERSDs, synucleinopathies, and, possibly, other conditions related. The key confirmation of this theory will only be obtained when future studies evaluate the feasibility of improving protein degradation to reduce storage in vivo.

## Figures and Tables

**Table 1:** Main comparison of aggregated proteins involved in PD, AATD, and HHHS.

Protein/Disease	α-Syn/PD	AAT/AATD	FG/HHHS
Native Structure	15 kDa Monomer *N*-terminal alpha-helix region, a central domain or NAC region and A C-terminal acidic tail	52 kDa Monomer Nine alpha-helices, two β-sheets and a reactive center loop	340 kDa triple fibrinogen Aα, Bβ, and γ chains Two lateral globular parts containing the *C*-terminus of Bβ and γ chains, a central nodule, containing the *N*-terminus of all chains
Polymerization steps	Monomer → dimer → oligomer → fibrils	Monomer → Dimer → Oligomer → Inclusion	Monomer → Oligomer → Protofibril → Fibril
Amyloid structure	Amyloid β-sheets in oligomers and fibrils	Amyloid β-sheets in dimers and oligomers	Amyloid fibril protein fibrinogen Aα
Inclusion bodies	LBs with more than 90 protein components	Inclusions with dense material and a clear halo in the periphery	Type I: Polygonal shape Type II: Ground glass appearance Type III: Eosinophilic globules, granular structures in the periphery
Inclusion proteins	α-syn, Tau protein, ubiquitin, neurofilament protein, β amyloid, among others	AAT M-Z and ZZ alleles	Mutated fibrinogen γ-chain
Organelles affected in the cell	α-syn aggregates can be found in all organelles	Only present in the ER	Only present in the ER
ER Stress response	UPR Chaperone activation PERK-dependent pathway	IL-6 and IL-8 protein production. Possible UPR activation. ER overload pathway	No available data
Organs affected	Across the brain tissue	Liver and lungs	Liver and lungs
Onset of disease	Chronic: Duplication/Triplication of *SNCA*: Symptoms from the age of 40 Idiopathic: From the age of 55	Chronic: Symptoms from early childhood	Chronic: Symptoms from early childhood or adulthood

**Table 4:** Targets for clinical strategies against FG in HHHS.

Hereditary Hypofibrinogenemia with Hepatic Storage				
Target	Strategy	Results *	Conclusions	Ref.
Autophagy	Expression of mutant γD domain of fibrinogen in yeast model	↑ Clearance of FG in ER by autophagy system	Aggregates of FG are cleared from the ER via the autophagic pathway.	[126]
Autophagy	Response to carbamazepine (CBZ) in patients with Fibrinogen storage disease (FSD).	↑ Autophagic activity by number of autophagocytic vacuoles ↓ Levels of alanine aminotransferase ↓ Caspase and cytokeratin fragments (M30 and M65).	CBZ enhanced autophagy and reduce aggregate-related toxicity in FSD	[138]
Proteolytic pathway	Treatment with ursodeoxycholic acid and α-tocopherol in children-patients with aguadilla HFSD	↓ Aspartate aminotransferase ↓ Alanine aminotransferase ↓ Serum bile acids ↓ Liver damage and fibrosis	This treatment has been proposed in children with HFSD and evidence of liver damage	[257]

* Arrows indicate increase (↑) or decrease (↓) of specific result.
